# Efficacy of Huangbai liniment for diabetic foot ulcers with multidrug-resistant organism infection: a randomized controlled multicenter trial

**DOI:** 10.3389/fendo.2026.1926211

**Published:** 2026-10-12

**Authors:** Ning Wang, Gang Wang, Min Zhou, Keren Zhang, Tong Zhou, Xuewan Wang, Guangyao Yang, Lijuan Deng, Tianyi Huang, Shang Ju

**Affiliations:** 1 Department of Peripheral Vascular, Dongzhimen Hospital of Beijing University of Chinese Medicine, Beijing, China; 2 Xiyuan Hospital of China Academy of Chinese Medical Sciences, Postdoctoral Research Station of China Academy of Chinese Medical Sciences, Beijing, China

**Keywords:** clinical trial, diabetic foot, infection, multidrug resistance, traditional Chinese medicine

## Abstract

**Background:**

Diabetic foot infection, particularly with multidrug-resistant organisms (MDROs), is a serious complication of diabetic foot ulcers (DFUs) that increases wound healing time and amputation risk. Prolonged use of broad-spectrum antibiotics can contribute to antibiotic resistance and limit future treatment options. Developing effective non-antibiotic antibacterial therapies is therefore urgently needed. Huangbai liniment (HB) is a standardized herbal product clinically used to treat diabetic foot infections, including methicillin-resistant *Staphylococcus aureusStaphylococcus aureus* (MRSA). This trial assessed the efficacy and safety of HB for DFUs with mild MDRO infection.

**Methods:**

This multicenter, open-label, randomized controlled trial was conducted in China. A total of 154 patients with DFUs and mild MDRO infection were randomly assigned to the HB group and the silver dressing (SD) group. Both groups received the same basic treatment for diabetic foot disease. The HB group received Tazi therapy (topical application of HB) for 2 weeks, while the control group was treated with SD. Primary outcome was the resolution of infection defined as a change from mild infection to no infection (using the Infectious Diseases Society of America Clinical Practice Guideline for the Diagnosis and Treatment of Diabetic Foot Infections). Secondary outcomes included the MDRO conversion rate, rate of completing ulcer healing, and reduction in ulcer size.

**Results:**

A total of 74 patients in the HB group and 63 patients in the SD group were included. Infection was resolved in 35 patients (47.3%) in the HB group versus 13 patients (20.6%) in the SD group (*p* = 0.001). Mixed-level logistic regression analyses showed that the HB group was associated with resolution of infection [odds ratio (OR) = 4.81; 95% confidence interval (CI): 2.01–12.48; *p* < 0.001]. However, there was no difference in rate of completing ulcer healing and reduction of ulcer size.

**Conclusion:**

Topical HB effectively facilitated mild infection resolution and MDRO conversion, with no adverse effects. HB is an effective and safe adjunctive therapy for short-term infection resolution in patients with DFU with mild MDRO infection. However, no evidence emerged for improved ulcer healing within 2 weeks. Longer follow-up is needed to assess wound healing outcomes.

**Clinical trial registration:**

https://www.chictr.org.cn/showproj.html?proj=66175., identifier ChiCTR2000041443.

## Background

Diabetic foot ulcer (DFU) is a serious complication of diabetes mellitus that leads to high rates of amputation and morbidity, poor quality of life, and a substantial financial burden on healthcare systems (1). The lifetime incidence of DFU is estimated to be 19% to 34% among people with diabetes (2). Once a patient with DFU develops infection, the risk of amputation will be increased by 155 times (3). Infection is one of the two leading causes of death for DFU (4). Meta-analysis showed that if the pathogen was a multidrug-resistant organism (MDRO), the risk and prevalence of amputation and death could increase as well (5). The Infectious Diseases Society of America (IDSA) Guideline for the Diagnosis and Treatment of Diabetic Foot Infections recommends that grade 2/mild infection should be treated with oral antibiotic therapy. However, the emergence of MDROs has rendered many antibiotics ineffective (6). Evidence is lacking to support the early use of broad-spectrum antibiotics against MDROs to promote diabetic ulcer healing, as such treatment may promote antimicrobial resistance (7), and antibiotic treatment failure can result in worsened bacterial infections (8). A key controversy in the treatment of mild diabetic foot infection (DFI) is whether local antimicrobial therapy can be effective in patients who are not eligible for systemic antimicrobial treatment. Therefore, there is a substantial need to develop effective and safe therapies that will benefit patients with DFU with MDRO mild infection.

Huangbai liniment (HB) is a traditional Chinese medicine preparation composed of Cortex Phellodendri (Huangbai), Scolopendridae (Wugong), *Lonicera japonica* Thunb. (Jinyinhua), *Taraxacum mongolicum* Hand.-Mazz. (Pugongying), and *Forsythia suspensa* (Lianqiao). HB has been shown to inhibit the growth of bacteria. Both *in vitro* and *in vivo* studies have proved that HB reduced the level of interleukin-17 (IL-17) and increased the level of transforming growth factor-β1 (TGF-β1) via activating the TGF-β and Nrf2 signaling pathway, inhibiting the expression of pro-inflammatory factors, and enhancing the generation of extracellular matrix during wound healing (9, 10). Cortex Phellodendri (Huangbai) is the sovereign herb in HB. Its primary active alkaloid, berberine, has been widely reported to exert anti-MDRO activity via its multi-target mechanisms, including membrane disruption and efflux pump inhibition, thereby acting against ESKAPE pathogens (11, 12). Although direct evidence for the whole HB formulation remains limited, the anti-MDRO activity of both Huangbai extract and its major component provides a reasonable rationale for the potential efficacy of HB against these pathogens. Topical administration of HB to patients with DFU has been proven to promote wound healing. However, these findings were derived from small-scale studies focusing on a single pathogen [methicillin-resistant *Staphylococcus aureus* (MRSA)], which limited the robustness and generalizability of the conclusions. It remains unclear whether patients with DFU with MDRO mild infection could benefit from HB therapy. Still, there is a need for a large-scale, well-designed, randomized controlled trial with clinical endpoints to determine the effects of HB on patients with DFU with MDRO mild infection. We hypothesized that treatment with HB for patients could be effective not only for patients with DFU with MRSA infection, but also for those with other MDROs. Therefore, the trial to assess the efficacy and safety of HB for DFU with MDRO mild infection was designed.

## Methods

### Study design

This was a prospective, open-label, randomized controlled trial conducted at nine clinical centers. This study followed the Consolidated Standards of Reporting Trials (CONSORT) reporting guideline for randomized clinical trials. The study protocol was approved by the Ethics Review Committee of Dongzhimen Hospital, Beijing University of Chinese Medicine (No. DZMEC-KY-2020-40) and conformed to the Declaration of Helsinki. Patients or their legally authorized representatives signed the informed consent. This trial was registered with the Chinese Clinical Trial Registry (ChiCTR2000041443).

### Participants

Patients were eligible if they (1) met the WHO diagnostic criteria for diabetic foot (1999); (2) met grade 2/mild infection (using the IDSA Clinical Practice Guideline for the Diagnosis and Treatment of Diabetic Foot Infections); (3) got MDR confirmed by bacterial culture; MDR was defined as acquired non-susceptibility to at least one agent in three or more antimicrobial categories (12); (4) had HbA1c ≤ 8%; (5) were female or male patients aged ≥18 years and ≤85 years; (6) had ankle brachial index (ABI) ≥ 0.4; (7) had a wound area between 1 cm × 1 cm and 10 cm × 10 cm; and (8) volunteered to participate in the experiment and sign an informed consent. Patients could be excluded if they (1) were allergic to HB or silver dressing (SD); (2) were treated with antibiotic in any form within 1 week before the treatment; (3) had severe heart, liver, or kidney dysfunction; (4) had ulcer not related to diabetes (venous ulcer, neoplastic ulcer, radiation-induced ulcer, simple arterial ulcer, etc.); (5) had a serum albumin level < 25 g/L; (6) had a hemoglobin level < 90 g/L; (7) had a platelet level below normal minimum; (8) were pregnant, breastfeeding, or had a pregnancy plan; (9) had cognitive dysfunction and failed to be fully informed; (10) were known allergies for ingredients in the investigational product or control product; (11) had participated in another clinical trial within 1 month; and (12) did not fulfill the study criteria or did not comply with the requirements judged by the investigators.

### Randomization

Individuals were randomized to the experimental and control groups at a 1:1 ratio. The randomization was performed by a statistician using the MagMinDA system. Stratified randomization was performed for ABI and bacterial classification. Bacterial classification was distinguished by Gram-positive and Gram-negative bacteria. The boundary value of ABI was 0.7, and values less than 0.7 indicate critical limb ischemia.

### Intervention

Both the HB group and the SD group received treatment for 2 weeks. All participants received standard care in accordance with current guidelines (13). Systematic treatment included smoking/alcohol cessation, diabetes diet, and control of blood pressure, blood sugar, and blood lipid level. Wound bed preparation included removal of inactivated tissue and incision of deep purulent cavity or sinus tract. The only variation was the randomization of the ulcers to have dressings. HB used in this trial was the commercially available Compound Huangbai Liquid Coating (Fufang Huangbaiye Tuji, manufactured by Shandong Hanfang Pharmaceutical Co., Ltd., China; Chinese medicine character: Z10950097), supplied in 100-mL glass bottles. The formulation consists of five herbal ingredients: Cortex Phellodendri, *F. suspensa*, *L. japonica* Thunb., *T. mongolicum* Hand.-Mazz., and Scolopendridae. The SD used in this study was Suprasorb^®^ A+Ag [calcium alginate silver fiber dressing, Lohmann & Rauscher (China) Medical Products Co., Ltd.].

In the HB group, the original solution of HB was soaked into 10 layers of sterile gauze (the thinnest layer of gauze) without dripping. The sterile gauze was then applied to cover the wound, extending 2 cm beyond the wound edge. If the wound was deep, sterile tweezers were used to insert the soaked sterile gauze into the wound cavity. The wound was soaked for 10 min, after which the gauze was removed and discarded. Subsequently, fresh sterile gauze soaked with HB was applied to cover the wound as described above. Meanwhile, an appropriate amount of dry sterile gauze (sufficient to avoid obvious penetration of HB) was placed over the soaked gauze, and the entire dressing was bandaged with a gauze bandage. The procedure was repeated once a day.

In the SD group, one piece of the SD (Suprasorb^®^ A+Ag) was taken. Using sterile tissue scissors, the dressing was trimmed to extend 2 cm beyond the wound edge. The dressing was then applied to cover the wound surface. If the wound was deep, the dressing was inserted into the wound cavity using sterile tweezers. Meanwhile, an appropriate amount of sterile gauze was placed over the SD, and the entire dressing was bandaged with a gauze bandage. The dressing was changed once a day. Every time the dressing was changed, the wound was carefully evaluated.

### Data collection

Data collection was performed online based on the clinical trial data management software eCDMS3.0 of Xiyuan Hospital, China Academy of Chinese Medical Sciences. Information obtained from patients was recorded in the dedicated case report forms by investigators, who received pre-recruitment standardized training regarding this trial’s standard operating procedures. Then, the clinical research coordinators performed data entry into electronic case report forms in eCDMS3.0. Information obtained from patients was recorded in the dedicated case report forms by investigators, who received pre-recruitment standardized training regarding this trial’s standard operating procedures. Subsequently, the clinical research coordinators performed data entry into electronic case report forms in eCDMS3.0.

### Efficacy outcomes

Because of the open-label design, the treating investigators who assessed efficacy outcomes were not blinded to group assignment. To mitigate potential detection bias, all assessors completed standardized training on the IDSA criteria before the trial, and all evaluations followed prespecified standard operating procedures at fixed time points.

Primary outcome was the resolution of infection defined as a change from grade 2/mild infection to grade 1/no infection (using the IDSA Clinical Practice Guideline for the Diagnosis and Treatment of Diabetic Foot Infections).

Secondary outcomes were conversion of MDRO, rate of completing ulcer healing, and reduction in ulcer size. Swab cultures accurately identified bacterial pathogens. MDRO was defined as acquired non-susceptibility to at least one agent in three or more antimicrobial categories. Conversion of MDRO was defined as bacterial pathogen no longer being multidrug resistant through the use of swab cultures. Ulcer healing was defined as achieving complete epithelialization. Ulcer size was measured using the eKare inSight™ system (a three-dimensional wound imaging and measurement device; eKare Inc., Fairfax, VA, USA). Efficacy outcomes were recorded at the 7th day and 14th day. The safety outcomes included any adverse events (AEs), aggravation of infection, and local allergic reactions. Aggravation of infection was defined as an aggravation from mild infection to moderate or severe infection. All safety outcomes that occurred from the point of withdrawal (day 0) to end of study participation were recorded.

### Statistical analysis

Statistical analyses were performed by independent statisticians using SPSS 27 or R 4.2.0. Continuous variables were presented as mean (standard deviation) and compared with *t*-test for normally distributed variables or presented as median (Q1 and Q3) and compared with Kruskal–Wallis tests for non-normally distributed variables. Counting data were analyzed by the chi-square test and Fisher exact test, while the ranked data were tested by Ridit analysis and Cochran–Mantel–Haenszel (CMH) analysis. Efficacy analyses were primarily performed on the full analysis set (FAS), defined as all randomized patients who received at least one dose of the study intervention and had at least one post-treatment efficacy assessment. Supportive per-protocol set (PPS) analyses were also conducted to assess the robustness of the findings. For confounding factors that were difficult to control or not controlled before treatment, the least square mean (lsmean) of analysis of covariance (ANCOVA) and its 95% confidence limit or logistic regression were used as covariates to determine the difference of curative effect between groups. Significance was set to a level of 0.05. Data analyses were completed by the Data Statistics Office of Clinical Pharmacology Research Institute of Xiyuan Hospital, CACMS.

## Results

### Search results and study characteristics

Recruitment was conducted from December 2020 to March 2022 at nine tertiary hospitals in China. A total of 154 patients were randomly assigned to the HB group and the SD group. In the HB group, seven participants withdrew due to COVID-19 prevention and control measures before any post-treatment assessment, while 10 in the SD group withdrew for the same reason and also had no post-treatment efficacy data. The FAS therefore comprised 74 patients in the HB group and 63 patients in the SD group. In this trial, the FAS was identical to the PPS, as all patients with at least one post-treatment assessment completed the full treatment period without major protocol deviations. Patients enrolled are outlined in **Figure 1**. The local and systemic characteristics were comparable between the two groups and there were no significant differences in demographic data, DFU characteristics, and comorbidities (**Table 1**). A total of 40 kinds of bacteria were isolated in this study. *Pseudomonas aeruginosa* was the most common MDRO in both groups, followed by *Staphylococcus aureusStaphylococcus aureus*, *Escherichia coli*, *Klebsiella pneumoniae*, and so on (**Table 2**).

**Figure 1:**
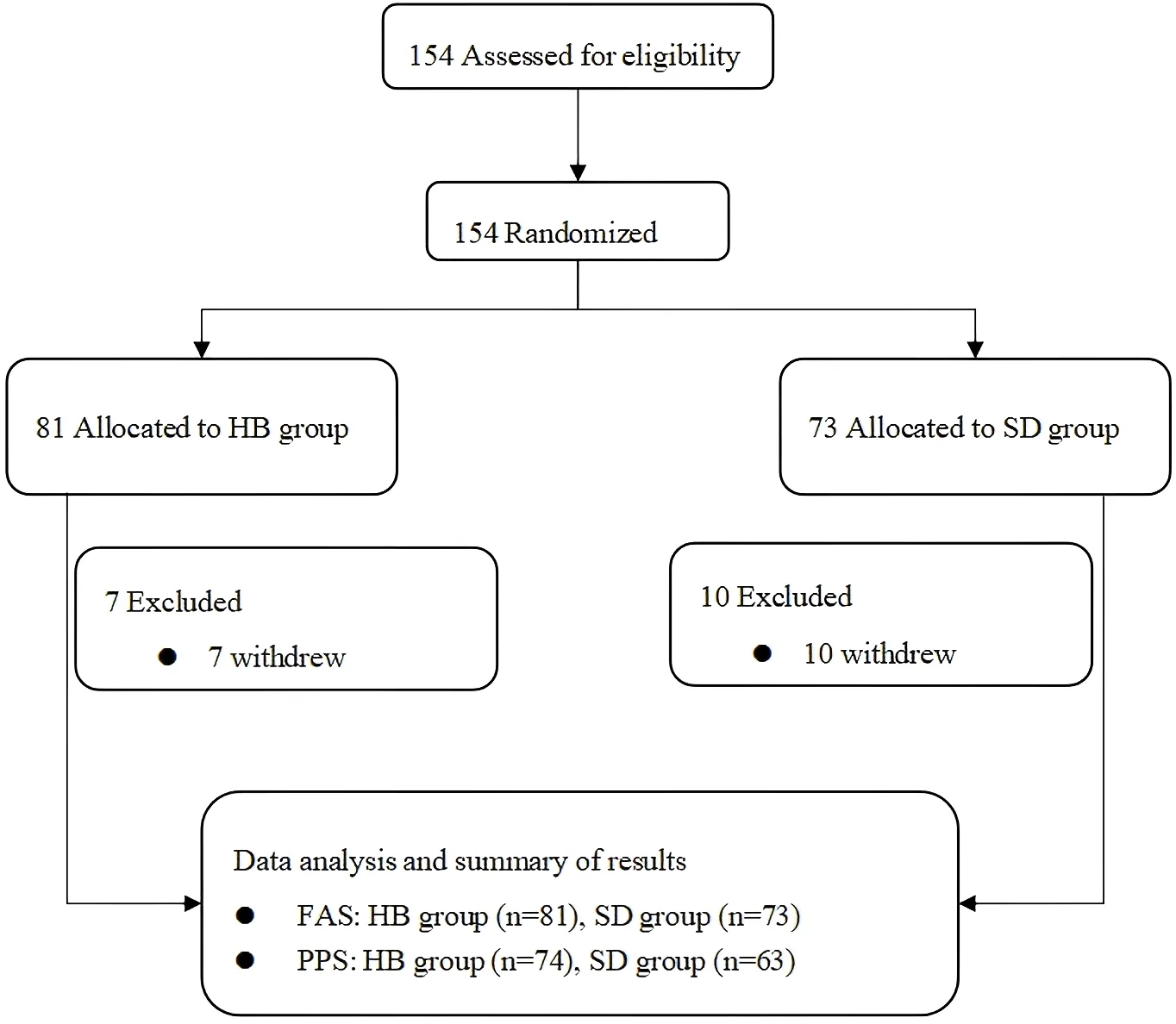
Flowchart of the participants (procedure) through the trial. FAS, full analysis set; PPS, per protocol set.

**Table 1:** Baseline characteristics of the HB group and the SD group.

Characteristic	HB group (*n* = 74)	SD group (*n* = 63)	*p*-value
Age, years, mean (SD)	65.5 (10.3)	63.8 (9.39)	0.34
Sex, female, *n* (%)	16 (21.6%)	17 (27.0%)	0.595
BMI, mean (SD)	23.9 (2.84)	23.9 (2.33)	0.853
Ulcer size, mean (SD)	9.53 (9.49)	7.78 (8.95)	0.269
ABI, mean (SD)	0.782 (0.179)	0.807 (0.224)	0.492
Hemoglobin, mean (SD)	118 (21.5)	120 (21.3)	0.508
Blood albumin, mean (SD)	36.5 (6.74)	35.7 (5.30)	0.439
Hypertension, *n* (%)	47 (63.5%)	39 (61.9%)	0.846
Neuropathy, *n* (%)	50 (67.6%)	46 (73.0%)	0.966
Retinopathy, *n* (%)	6 (8.1%)	9 (14.3%)	0.283
Nephropathy, *n* (%)	2 (2.7%)	5 (7.9%)	0.347
Dyslipidemia, *n* (%)	0	0	–
Ischemic heart disease, *n* (%)	27 (36.5%)	31 (49.2%)	0.133
Ischemic cerebrovascular disease, *n* (%)	18 (24.3%)	10 (15.9%)	0.169

SD, standard deviation; BMI, body mass index; ABI, ankle brachial index.

**Table 2:** Baseline pathogenic bacteria distribution in patients with DFU.

Bacteria	HB group (*n* = 74)	SD group (*n* = 63)
*Pseudomonas aeruginosa*, *n* (%)	16 (15.1%)	23(23.5%)
*Staphylococcus aureusStaphylococcus aureus*, *n* (%)	10 (9.4%)	13 (13.3%)
*Escherichia coli*, *n* (%)	14 (13.2%)	6 (6.1%)
*Klebsiella pneumoniae*, *n* (%)	4 (3.8%)	11 (11.2%)
*Serratia marcescens*, *n* (%)	7 (6.6%)	7 (7.1%)
*Acinetobacter baumannii*, *n* (%)	9 (8.5%)	5 (5.1%)
*Proteus mirabilis*, *n* (%)	3 (2.8%)	9 (9.2%)
*Enterobacter cloacae*, *n* (%)	5 (4.7%)	5 (5.1%)
*Staphylococcus epidermidis*, *n* (%)	5 (4.7%)	5 (5.1%)
*Proteus vulgaris*, *n* (%)	4 (3.8%)	2 (2.0%)
*Citrobacter freundii*, *n* (%)	4 (3.8%)	2 (2.0%)
Others, *n* (%)	25 (23.6%)	10 (10.2%)
Total	106 (100%)*	98(100%) *

*The total number of isolates exceeds the number of patients with polymicrobial infections.

### Outcomes

#### Primary outcome

Mild infection resolved in 35 patients (47.3%) in the HB group versus 13 patients (20.6%) in the SD group (*p* = 0.001) (**Table 3**). Mixed-level logistic regression analyses, adjusting for age, sex, body mass index (BMI), ulcer size, ABI, and neuropathy, showed that the HB group was associated with resolution of infection [odds ratio (OR) = 4.81, 95% confidence interval (CI): 2.01–12.48, *p* < 0.001] (**Table 4**). Wound (different parts including toe, dorsum, sole, and heel) photos from seven HB-treated patients and seven SD-treated patients are presented in **Figure 2**.

**Table 3:** Primary outcome and secondary outcomes.

Outcome	HB group (*n* = 74)	SD group (*n* = 63)	*p*-value
Primary outcome			
Resolution of infection, *n* (%)	35 (47.3%)	13 (20.6%)	0.001
Secondary outcomes			
Conversion of MDRO, *n* (%)	15 (20.3%)	8 (12.7%)	0.237
Ulcer healed, *n* (%)	58 (78.38%)	44 (69.84%)	0.406
Reduction of ulcer size	2.12 (9.37)	1.56 (3.97)	0.668

MDRO, multidrug-resistant organism.

**Table 4:** Odds of resolution of infection, based on treatment group (HB vs. SD).

Variable	OR	95% CI	*p*-value
HB group	4.808	2.005–12.478	<0.001**
Sex (female)	1.278	0.478–3.576	0.630
Age	0.99	0.944–1.037	0.682
BMI	1.046	0.85–1.289	0.672
Ulcer size	1.02	0.968–1.081	0.466
ABI	0.736	0.059–9.569	0.811
Neuropathy (not)	0.717	0.273–1.889	0.496

BMI, body mass index; ABI, ankle brachial index.

**Figure 2:**
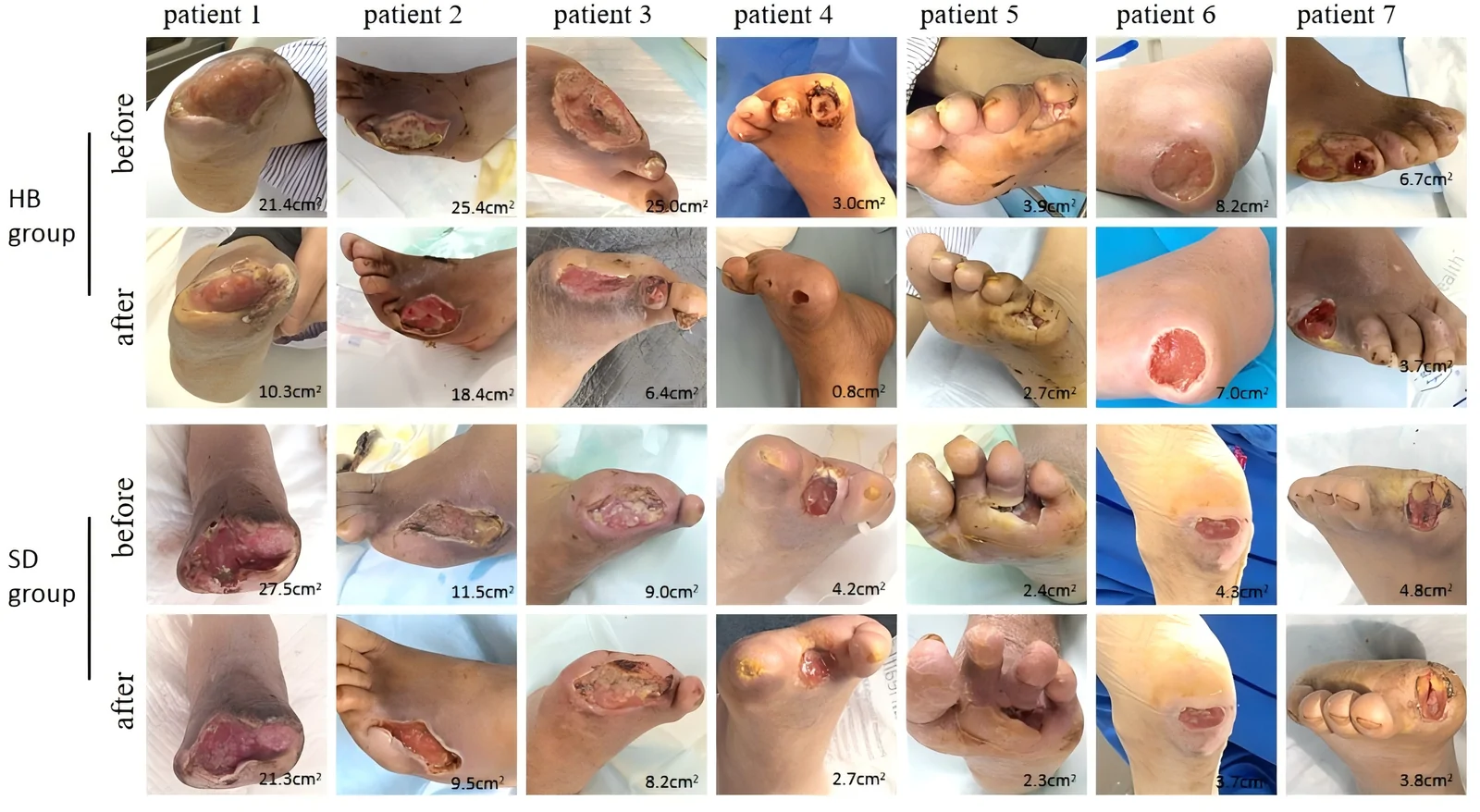
Wound photos from seven HB-treated patients and seven SD-treated patients.

#### Secondary outcomes

MDRO conversion occurred in 15 patients (20.3%) in the HB group and 8 patients (12.7%) in the SD group (*p* = 0.237). There was no difference in rate of completing ulcer healing and reduction of ulcer size (**Table 3**). However, mixed-level logistic regression analyses, adjusting for age, sex, BMI, ulcer size, ABI, and neuropathy, showed that the HB group was associated with resolution of infection (OR = 4.81, 95% CI: 2.01–12.48, *p* < 0.001) (**Table 4**).

#### Safety outcomes

No AE occurred during the study. Neither aggravation of infection nor significant local allergic reaction occurred in the two groups.

## Discussion

HB, made from traditional Chinese medicine, is a pretty good topical medicine and has been shown to accelerate the wound healing process (14). Although several studies have reported the efficacy of HB for DFU, they were limited by small sample sizes and a focus on a single pathogen (MRSA) among MDROs (15, 16). Thus, a large, multicenter, randomized controlled study to evaluate the efficacy and safety of HB for patients with DFU with MDRO infection was performed. In this study, HB was compared with SD in addition to standard of care. The assessment of HB for treating patients with DFU with MDRO infection was a multidimensional evaluation. In our study, we evaluated the curative effect of HB in the following several aspects. Firstly, resolution of infection rate was calculated to be the primary outcome. Secondly, conversion of MDRO, ulcer healing, and reduction of ulcer size performed to assess the efficacy of HB were considered secondary outcomes. Finally, any AEs and clinically meaningful changes in vital signs or laboratory parameters during the trial period were recorded to assess the safety of HB. Taken together, the multidimensional evaluation system aimed to assess the efficacy and safety of HB for patients with DFU with MDRO infection more comprehensively. SD was considered to be an adequate comparator because (1) the clinical and antimicrobial efficacy of SD has been proven in the management of DFI (17), and (2) silver has a broad-spectrum antimicrobial activity including inactivating intracellular enzymes, inhibiting bacterial cell respiration, and destroying cell membranes. Meta-analysis showed that SDs enhanced DFU healing rate and improved the infection resolution rate (18). Silver is supposed to fight against multidrug-susceptible and multidrug-resistant strains. MDRO increase has revived the use of silver as an alternative antibiotic-independent antimicrobial (19). This growing interest in metal-based antimicrobial dressings, including copper-impregnated alternatives, reflects the broader search for antibiotic-independent strategies to combat MDRO infections in chronic wounds (20, 21).

In terms of clinical application, our results demonstrated that HB effectively helped achieve resolution of infection. Mixed-level logistic regression analyses showed that compared with SD, HB was associated with resolution of mild infection and conversion of MDRO in a clinical setting. Once infection is involved in DFU, debridement is considered to reduce bacterial load and eradicates bacterial biofilm. The most effective debridement methods are those that rapidly, aggressively, and comprehensively remove pus, slough, or necrotic tissue. Affected by compromised immune systems and/or poor vascular perfusion, the signs and symptoms of infection may be covert and subtle, and debridement can proceed only after a comprehensive assessment of the individual and their wound. Commonly, slough may be firmly attached to the granulation tissue. In this situation, sharp debridement can easily cause local bleeding. HB has a significant clinical advantage in removing slough firmly attached to the wound (typical photos are shown in **Figure 2**). Unlike sharp debridement, which may cause bleeding, HB application can gradually remove firmly attached slough without pain or bleeding, similar to the slow but steady action of a silkworm consuming a leaf. Debridement like silkworm eating can remove infected tissue without pain or bleeding. HB application in removing slough firmly attached to the wound can be considered as debridement like silkworm eating. In this trial, reduction of ulcer size indicated that the average value of the HB group is higher than that of the SD group, but the result was not significant. This suggests that there is insufficient evidence that HB promotes ulcer healing within the observation period, which is inconsistent with previous findings (22). This may be related to the subjects infected or not and the short observation period not yet reaching the epithelial growth stage.

In terms of mechanism, non-healing DFU is related to persistent inflammation, intense bacterial infection, and impaired angiogenesis (23). Persistent inflammation, manifested by the presence of the high level of pro-inflammatory cytokines, is considered a major contributor to healing impairment in DFU. *In vitro* and *in vivo* experiments have shown that HB and its major component berberine can both significantly repress the IL-17 signaling pathway, a key pathway that induces inflammation (10).

While HB significantly improved infection resolution within the 2-week treatment period, no statistically significant difference was observed in ulcer healing rates between the two groups. This discrepancy may be attributable to the different time courses of these two clinical endpoints. Infection resolution is primarily an antimicrobial event that can occur relatively quickly once effective antibacterial activity is established. Wound healing, in contrast, is a multi-stage biological process that normally takes weeks to months to complete (24). In patients with diabetes, wound healing is often delayed. Persistent inflammation and impaired transition from the inflammatory to the proliferative phase are well-recognized features of diabetic wounds (25). MDROs further exacerbate this delay by forming biofilms and expressing virulence factors that directly disrupt the inflammatory and proliferative phases (26). Consequently, even after clinical infection is controlled, the wound bed often remains in a pro-inflammatory, pro-protease microenvironment that is not yet conducive to rapid epithelialization (27). Moreover, a 2-week observation period may be too short to capture meaningful differences in healing rates. Therefore, the anti-inflammatory properties of HB may require a longer follow-up period to translate into improved healing. Future trials with extended observation periods (e.g., 4 to 12 weeks) are warranted.

Strict quality control criteria were performed in this study to ensure the reliability and reproducibility of the results. For the intervention of the study, we have established the standard procedures for both the HB group and the SD group including the amount of dressing and frequency of the treatment. To ensure the standardization of the treatment, participation enrollment and intervention were completed by clinical physicians who have received standardized training. For data collection and management, the information was recorded in the dedicated case report forms by investigators who received pre-recruitment standardized training. Every case report form was made electronic and converted to PDF, which could be stored in persistent and non-editable storage media at medical centers. For data monitoring, the Steering Committee and the contract research organization were responsible for overseeing the trial operations and the project data manager and the monitor regularly performed data monitoring and data quality control.

### Limitations

This study has several limitations. First, the open-label design is a major limitation. Because both investigators and patients were aware of the treatment allocation, performance bias and detection bias cannot be excluded. The primary outcome—resolution of infection—involves clinical judgment of local signs. Although the assessors used standardized IDSA criteria, which may partly mitigate detection bias, the lack of blinding could have influenced the assessment, potentially inflating the treatment effect estimate. Therefore, the observed OR of 4.81 should be interpreted with caution. Future studies should employ blinding of outcome assessors. Second, the observation period was only 2 weeks, which may have been too short to detect differences in ulcer healing. Third, the low event rate for complete ulcer healing precluded multivariable adjustment. Fourth, the wide CI for MDRO conversion indicates imprecision; thus, this result is exploratory. Fifth, affected by the COVID-19 pandemic, 17 participants discontinued due to isolation, which may introduce attrition bias. Finally, while we adjusted for predefined covariates including bacterial type to increase precision, residual confounding cannot be excluded. Future studies with larger sample sizes, a longer follow-up, and a blinded design are needed to confirm these findings.

## Conclusion

In conclusion, our study revealed that HB effectively resolved mild infection within a 2-week treatment period in patients with DFU with MDRO infection, with no adverse effects observed. However, no statistically significant difference was found in ulcer healing rates or ulcer size reduction between the HB and SD groups, which may be attributable to the short observation period not yet reaching the epithelialization phase. This is the largest randomized controlled trial to date evaluating HB in this population. These findings suggest that topical HB can be an effective and safe adjunctive therapy for achieving short-term infection control in patients with DFU with mild MDRO infection. Given the short follow-up duration, the results should be interpreted with caution, and future studies with longer observation periods are warranted to determine whether infection resolution translates into improved ulcer healing.

## Data Availability

The raw data supporting the conclusions of this article will be made available by the authors, without undue reservation.
